# Oxidative stress induction by crude extract of *Xylaria* sp. triggers lethality in the larvae of *Aedes aegypti* (Diptera: Culicidae)

**DOI:** 10.1590/0037-8682-0373-2021

**Published:** 2022-04-29

**Authors:** Maria Beatriz Silva Costa, Rejane de Castro Simões, Márcia de Jesus Amazonas da Silva, André Correa de Oliveira, Leonard Domingo Rosales Acho, Emerson Silva Lima, Wanderli Pedro Tadei, Helder Lopes Teles, Camila Martins de Oliveira

**Affiliations:** 1 Universidade Federal do Amazonas, Instituto de Ciências Exatas e Tecnologias, Itacoatiara, AM, Brasil.; 2 Instituto Nacional de Pesquisas do Amazonas, Laboratório de Malária e Dengue, Manaus, AM, Brasil.; 3 Universidade Federal do Amazonas, Faculdade de Ciências Farmacêuticas, Manaus, AM, Brasil.; 4 Universidade Federal de Rondonópolis, Instituto de Ciências Exatas e Naturais, Rondonópolis, MT, Brasil.

**Keywords:** Cytotoxicity, Carbonyl, Dengue, TBARS, Endophytic fungus

## Abstract

**Background:**

*Aedes aegypti* is currently controlled with synthetic larvicides; however, mosquitoes have become highly resistant to these larvicides and difficult to eradicate. Studies have shown that insecticides derived from fungal extracts have various mechanisms of action that reduce the risk of resistance in these mosquitoes. One possible mechanism is uncontrolled production of reactive oxygen species (ROS) in the larvae, which can cause changes at the cellular level. Thus, the crude extract of *Xylaria* sp. was evaluated to investigate the oxidative effect of this extract in *A. aegypti* larvae by quantifying the oxidative damage to proteins and lipids.

**Methods:**

The larvicidal potential of the crude extract of *Xylaria* sp. Was evaluated, and the extract was subsequently tested in human lung fibroblasts for cytotoxicity and ROS production. ROS level was quantified in the larvae that were killed following exposure to the extract in the larvicide test.

**Results:**

The crude extract of *Xylaria* sp. Caused cytotoxicity and induced ROS production in human lung fibroblasts and *A. aegypti* larvae, respectively. In the larvicide trial, the extract showed an LC_50_ of 264.456 ppm and an LC90 of 364.307 ppm, and was thus considered active. The extract showed greater oxidative damage to lipids and proteins, with LC_90_ values of 24.7 µmol MDA/L and 14.6278 ×10^-3^ nmol carbonyl/ mg protein, respectively.

**Conclusions:**

Crude extracts of *Xylaria* sp. induced oxidative stress that may have caused the mortality of *A. aegypti* larvae.

## INTRODUCTION

The mosquito *Aedes aegypti* is the main vector of dengue, chikungunya, and Zika virus^1^ ^,^ ^2^, which are considered serious public health problems in Brazil and worldwide. The tropical regions of Brazil has the highest incidence of these diseases^2^. The northern region had the highest number of probable cases of Zika (919 cases; 39.2%) of the total cases in Brazil^3^. As no vaccine against the aforementioned diseases is available, the most effective mechanism for prevention is to control the vectors (mosquitoes and larvae). Thus, the integrated vector control measure defined by the World Health Organization (WHO)^4^ includes inspection, environmental management, biological control, chemical control using insecticides and repellents, traps, and insecticide resistance management. The biological control of mosquitoes involves the use of various predators, aquatic invertebrates, fungal and bacterial pathogens, nematode parasites, and fish that feed on larvae^5^. Chemical control is currently carried out by synthetic larvicides derived from organophosphorus compounds, carbamates, and pyrethroids; however, they cause great environmental damage, are costly^6^, and have been causing high resistance rates in *A. Aegypti* ^7^ *.*

Owing to the resistance of mosquitoes and the low effectiveness and high cost of programs to control the vectors of these diseases^8^ ^-^ ^10^, new strategies are needed to combat vectors using mechanisms that are less polluting, less toxic, and pose less risk to human health^11^ ^,^ ^12^. Some studies involving natural bioactive products made from endophytic fungi have revealed promising alternatives^13^. Endophytic fungi are microorganisms that inhabit the internal parts of host plants in all or part of their life cycle and produce a vast amount of bioactive metabolites^14^ ^,^ ^15^.

According to Aury^7^, larvicides and insecticides from endophytic fungal extracts are widely used today and have an advantage over synthetic compounds, given their various action mechanisms, which reduces the risk of resistance in mosquitoes^17^ ^,^ ^16^. According to the literature^17^ ^,^ ^18^, an example of the action mechanism of endophytic fungi is the release of reactive oxygen species (ROS), which results in cellular oxidative damage in insects and larvae^17^ because ROS are free radicals that can cause cellular toxicity when antioxidant enzymes are produced in less quantity than ROS^18^.

Although many studies on the metabolites of fungal endophytes have been conducted, only one has been reported^19^, in which endophytes of the genus *Xylaria* showed a larvicidal activity against *A. aegypti* ^20^. *Xylaria* endophytes belong to the family Xylariaceae (Sordariomycetes, Xylariales), which has at least 85 genera and probably more than 1,000 species^21^, and can produce metabolites with a wide spectrum of biological activities^20^ ^,^ ^22^. Thus, this study aimed to evaluate the larvicidal activity of the raw extract of *Xylaria* sp. against third-instar larvae of *A. aegypti*, and to verify the possible entomotoxic effects of this extract.

## METHODS

### Plant material

The endophytic fungus *Xylaria* sp. was isolated from the plant species *Passovia Stelis* (L.) Kuijt (Lorantahaceae), which was collected from the campus of the Federal University of Amazonas (3°08′57″S, 58°26′38″W), in the city of Manaus, Amazonas, Brazil. A voucher specimen (No. 11422) was deposited in the herbarium of the university.

### Isolation and identification of the fungus

The endophytic fungus was isolated according to the method described by Maier^23^ and Souza^24^. Fungal isolates were identified by grouping their macromorphological and micromorphological similarities. The endophytic fungi were preserved in distilled water^25^.

### Preparation of Xylaria sp. raw extract

*Xylaria* sp. was harvested and transferred to 35 Erlenmeyer flasks containing 300 mL of potato dextrose broth and maintained at a controlled temperature of 28 °C for 28 days. After fermentation, the medium was filtered to remove the visible mycelium and subjected to liquid/liquid partition with ethyl acetate evaporated in a rotary evaporator. The resulting raw extracts were subsequently subjected to larvicide testing.

### Larvicidal test

#### Mosquito breeding

The eggs of *A. aegypti* were obtained from a colony kept in an insectarium at the Laboratory of Malaria and Dengue of the National Amazon Research Institute. The eggs were then immersed in water until hatching. For bioassays, the resulting larvae were fed crushed larval food until the third instar phase under the growth conditions described by Medeiros et al.^26^: temperature, 26 ± 2 °C; humidity, approximately 80%, photoperiod, 12 h.

#### Selective larvicidal bioassay

Five disposable cups with a capacity of 50 mL containing 9.8 mL of water were used for the selective bioassay. Briefly, 10 third-instar larvae of *A. aegypti* were introduced into each cup. Next, 100 µL of *Xylaria* sp. extract diluted in dimethyl sulfoxide (DMSO) was pipetted separately into each cup at concentrations of 500, 250, 125, 62.5, and 31.25 ppm, followed by 100 µL of crushed larval food. Each bioassay was performed in triplicates. DMSO (1%) was used as a negative control. The final volume of each cup was 10 mL. Bioassay readings were taken at 24, 48, and 72 h after the exposure of the larvae to the extract. 

#### Dose-response bioassay

Dose-response bioassays were performed according to the WHO protocol^3^. The five concentrations used in this assay were developed from the lowest concentration that caused at least 50% mortality of the larvae in the selective bioassays. The test was performed in quintuplicates. Data were analyzed by one-way ANOVA (*p* ≤ 0.05) using the GraphPad Prism software (version 6.0; San Diego, CA, USA) and expressed as mean (%) ± standard deviation. Lethal concentrations (LC_50_ and LC_90_) and confidence intervals (CI = 95%) were calculated using Probit analysis in the Poloplus software version 1.0 (LeOra Software, Berkeley, CA)^27^.

### *In vitro* cytotoxicity

Cytotoxicity testing of *Xylaria* sp. extract was performed according to the methodology described by Ahmed et al.^28^. Resazurin (AlamarBlue®) and MRC-5 cells (ATCC-CCL-171-human lung fibroblasts) were used to evaluate the toxicity of the extract in humans if used as a larvicide. Doxorubicin (20 µg.mL^-1^) was used as a positive control, and DMSO (0.1%) was used as a negative control. Fluorescence was measured at 570 nm using an Elisa Microplate reader (DTX-800; Beckman Coulter). Data were analyzed by a two-way ANOVA test (*p* ≤ 0.05) using GraphPad Prisma version 6.0, and were used to calculate the IC_50_ of the extract.

### Determination of ROS levels using DCFH-DA

ROS levels in MRC-5 cells following treatment with *Xylaria* sp. extract were determined using 2′,7′- dichlorohydrofluorescein diacetate (DCFH-DA) according to the method described by Eruslanov and Kusmartsev^29^. Paclitaxel (256 µg) mL^-1^) and hydrogen peroxide (17 µg. mL^-1^) were used as positive controls, and buffered saline solution (PBS) was used as a negative control. Cell fluorescence was determined at 570 nm using an Elisa Microplate reader (Dx800 multimode detector; Beckman Coulter). Data were analyzed by two-way ANOVA (p≤ 0.05) using GraphPad Prisma version 6.0.

### Lipid and protein oxidative damage assay

#### Homogenisation of larvae

Larvae of *A. aegypti* subjected to the larvicide test at lethal doses of 50% and 90% were cold-macerated in a pestle and mortar with 10 mL of phosphate buffer (pH 7.3, 75 mM for each 1 g of larvae). The homogenate was centrifuged at 15,000 rpm for 30 min at 4 °C. The supernatant was used to quantify oxidative damage to proteins and lipids^30^. 

#### Lipid oxidative damage

Lipid oxidative damage was measured using malondialdehyde (MDA), in accordance with the technique described by Ohkawa and et al.^31^. The total MDA level in the samples was calculated as the ratio of sample absorbance to that of a standard MDA solution (1,1,3,3-tetrahydroxypropane) multiplied by the standard solution concentration (concentration curve). Larval homogenate and phosphate buffer (75 mM) were used as negative controls. The results are expressed in μmol/L. The data were subjected to statistical analysis by two-way ANOVA with Dunnett’s test (*p* ≤ 0.05) for the control using GraphPad Prism (version 6.0). All experiments were performed in triplicate.

#### Oxidative damage to proteins

The protein oxidative damage test was performed according to an adaptation of the methodology initially described by Levine et al. (1990)^32^ and Lowry (1951)^33^, in which oxidative damage to proteins was measured by quantifying carbonyl groups based on reaction with 2,4-dinitrophenyl hydrazine (DNPH) prepared in HCl 2.5 M^32^. Protein content was determined using the Lowry method with BSA as a standard^33^. Larval homogenate and HCl 2.5 M were used as negative controls. All experiments were performed in triplicates. Statistical tests were performed using the two-way ANOVA test with Dunnett’s test (*p* ≤ 0.05) concerning the control using GraphPad Prisma version 6.0. All experiments were performed in triplicate.

## RESULTS

### Larvicidal test: selective and dose-reactive bioassays

According to the results of the selective bioassay, *Xylaria* sp. extract resulted in a satisfactory mortality rate of over 50% in *A. aegypti* larvae, but only concentrations between 250 and 500 ppm exhibited larvicidal activity. Thus, the concentrations used in the subsequent dose test were in the range of 250-500 ppm (400, 375, 350, and 325 ppm) (Table 1).

**TABLE 1:** Results of dose-reactive bioassays of different concentrations of *Xylaria* sp. extract in *A. aegypti* larvae.

Treatment	Concentration (ppm)	***n* (samples)**	Mortality (%)		
			24 h	48 h	72 h
*Xylaria* sp.	400	100	91	5	0
	375	100	80	9	2
	350	100	69	17	0
	325	100	61	13	3
	300	100	49	25	2
	275	100	35	13	7
DMSO	1 (%)	100	0	0	0

The mortality rates at 24, 48, and 72 h after exposure revealed that the highest mortality rate occurred at 24 h after treatment of *Xylaria* sp. extract a concentration of 400 ppm, with a mortality rate of 91% (Table 1). This finding revealed a satisfactory larvicidal activity of *Xylaria* sp. extract against the of *A. aegypti* larvae, as according to the WHO^34^, an extract is considered active if the mortality rate is greater than 80%. 

According to the probit analysis, the LC_50_ value of *Xylaria* sp. extract was 264.456 ppm, and the LC_90_ value was 364.307 ppm, with a significance level of 95% (Table 2). The parametric test (χ^2^) showed no significant difference between the two doses (LC_50_ and LC_90_), as both concentrations caused mortality in the larvae. Thus, the concentrations corresponding to the LC_50_ and LC_90_ are expected to cause 50% and 90% mortality of the larvae, respectively.

**TABLE 2:** LC_50_ and LC_90_ values of *Xylaria* sp. extract against *A. aegypti* larvae after 72 h of treatment. No mortality was observed in the negative control (DMSO) group.

Extracts	Concentration (ppm)^a^	Mortality (%) ± SD^b^	LC_50_ (ppm) (LCL-UCL)^c^	LC_90_ (ppm) (LCL-UCL)^d^	Regression equation	χ^2 e^
*Xylaria* sp.	400	96.7 ± 0.0	264.456 (245.835-277.284)	364.307 (350.9-384.617)	y = -17,315+9,212x	3.263 ns ^f^
	375	93.3 ± 1.7				
	350	82.2 ± 2.3				
	325	77.8 ± 2.3				
	300	76.7 ± 2.6				
	275	53.3 ± 4.6				
	0	0.0 ± 0.0				

^a^Concentration in ppm; ^b^SD: Standard deviation; ^c^LC_50_ **:** Lethal concentration that kills 50% of the larvae; ^d^LC_90_ **:** Lethal concentration that kills 90% of the larvae; ^e^χ^2:^ Chi-square; ^f^n. s: Not significant (α = 0.05) (means that the data were fitted to the software without the need for adjustments.

### *In vitro* test in cells: cytotoxicity and ROS production

**FIGURE 1:**
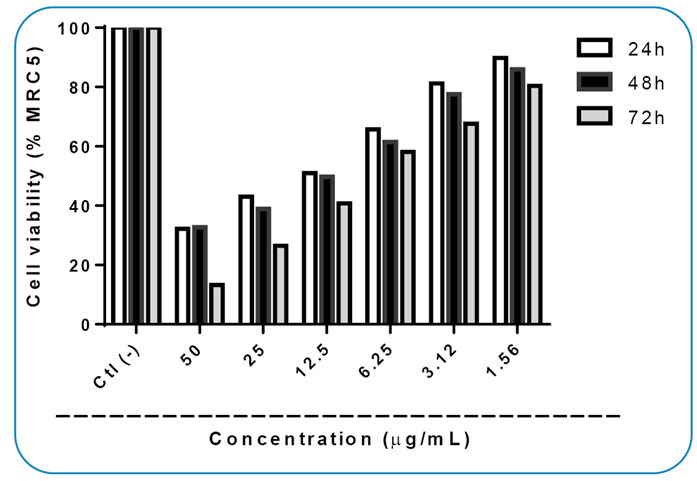
Cell viability of MRC-5 fibroblasts treated with *Xylaria* sp. extract at different concentrations for 24, 48, and 72 h. Data are expressed as mean ± SEM (n = 3). P<0.05 is significant compared to the negative control (Ctl(-)), according to two-way ANOVA with Dunnett’s test.

The *Xylaria* sp. extract was cytotoxic at a concentration of less than 50 µg.mL^-1^ (Figure 1), capable of inhibiting the growth of more than 50% of cells, compared with the standard drug doxorubicin at a concentration of 20 µg.mL^-1^. Considering the mass death of MRC-5 cells, ROS level was also evaluated in these cells to verify whether the death of these cells was due to increased ROS production.

Cytotoxicity assay was conducted using different concentrations (50, 25, 12.5, 6.25, 3.12, and 1.56 µg.mL^-1^) of *Xylaria* sp. extract (Figure 1), and the results showed that the higher the dose, the higher the cytotoxicity of this extract. Using the concentration curve, the IC_50_ of the extract was calculated to be 6.835, 5.940, and 11.38 µg.mL^-1^ after 24, 48, and 72 h of exposure, respectively.

**FIGURE 2:**
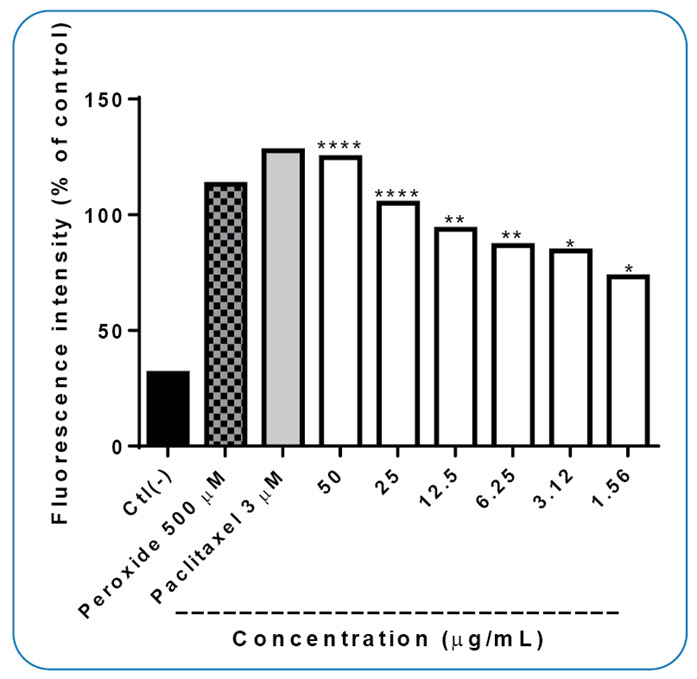
ROS concentration curve in MRC-5 human lung fibroblasts treated with *Xylaria* sp. extract. Data are expressed as mean ± SEM (n = 3). P<0.0001 is significant compared to the negative control (Ctl (-)), according to two-way ANOVA with Dunnett’s test. The standard used was 3 µm paclitaxel (256 µg.mL^-1^).

ROS assay was also performed using different concentrations (50, 25, 12.5, 6.25, 3.12, and 1.56 µg.mL^-1^ **)** of *Xylaria* sp. extract (Figure 2). The results revealed that *Xylaria* sp. extract at all tested concentrations induced ROS production compared to the negative control. However, the highest ROS level was observed in cells treated with *Xylaria* sp. extract at 50 and 25 µg.mL^-1^; moreover, ROS production decreased as the extract concentration decreased.

### Evaluation of oxidative damage in *A. aegypti* larvae

**FIGURE 3:**
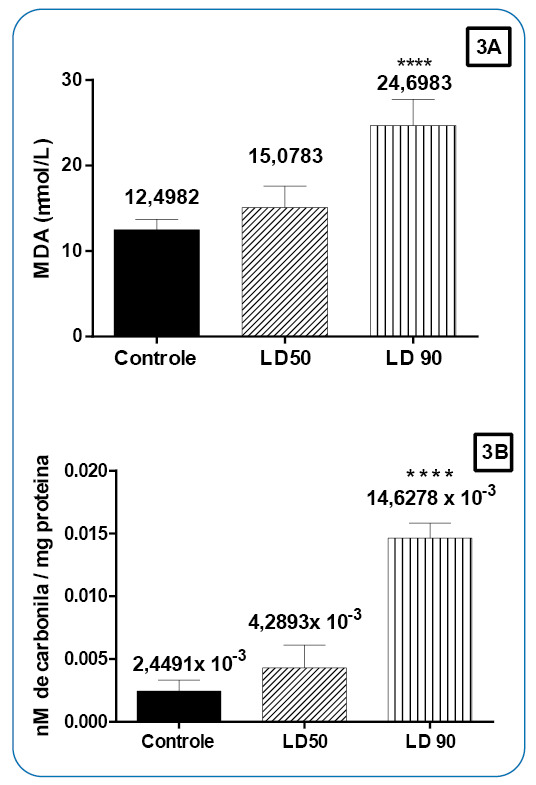
**A)** Bar graph illustrating lipid peroxidation in *A. aegypti* larvae treated with *Xylaria* sp. extract at the lethal dose 50 (LD_50_), Xylaria sp. extract at the lethal dose 90 (LD_90_), and 1% DMSO (control). Data are expressed as MDA levels in mmol/L in the samples. **B)** Bar graph illustrating protein oxidation in *A. aegypti* larvae treated with *Xylaria* sp. extract at the LD_50_, *Xylaria* sp. extract at the LD_90_, and 1% DMSO (control). Data are expressed in nM carbonyl/mg protein.

Statistical analysis revealed damage to the lipids of the larvae treated with *Xylaria* sp. extract, as the MDA levels were significantly (*p* <0.0001) different between the larvae treated with *Xylaria* sp. at the LC_90_ and the control (macerated larvae not exposed to the extract), with MDA levels almost twice as high (24.7 µmol/L) as that in the negative control (12.5 µmol/L). Larvae subjected to *Xylaria* sp. extract at the LC_50_ showed no significant difference from the control, with a confidence limit of 95% (Figure 3A). These results were corroborated by the results obtained in the ROS production test of the *in vitro* extract, which showed an increase in total ROS (Figure 2).

Protein oxidative damage was also examined in *A. aegypti* larvae, and the results were also subjected to statistical analysis. Carbonyl levels were significantly (*p* <0.0001) different between the larvae exposed to the extract at LC_90_ (14.6278 ×10^-3^ nmol carbonyl/mg protein) and the control (2.4491 ×10^-3^ nmol carbonyl/mg protein). Carbonyl levels were also significantly (*p* <0.0018) different between the larvae treated with the extract at the LC_50_ (4.2893 ×10^-3^ nmol carbonyl/ mg protein) and the control. These values had a confidence limit of 95%, as shown in Figure 3B. Thus, these findings corroborate the protein oxidative damage assay results.

## DISCUSSION

According to previous studies, endophytic fungi are considered potential producers of bioactive chemicals^35^, especially those of the genus *Xylaria*, which includes several species of fungi found in plants. Moreover, they produce a wide variety of metabolites with different chemical structures, such as cytochalasins, terpenoids, alkaloids, coumarins, and benzoquinones, with a range of biological activities, such as phytotoxic, antifungal, antimalarial, and antibacterial activities^36^. 

The results of the present study support that *Xylaria* sp. extract can be an alternative source of larvicide for *A. aegypti* control, showing comparable efficacy to other extracts previously reported^37^ (6.6% mortality at a concentration of 250 µg.mL^-1^). Moreover, as a product derived from a biological source, *Xylaria* sp. extract is more beneficial than the synthetic larvicide temephos in terms of sustainability, action mechanism, and toxicity.

The results of lipid peroxidation analyses (Figure 3A) could be related to ROS, which might have caused membrane damage in the larvae^38^. In addition, as shown in Figure 3A, the control larvae also showed oxidative damage, as indicated by the MDA levels, possibly because the larvae were macerated alive and underwent stress until death, and any death process can lead to oxidative damage.

Lipid peroxidation is a chain reaction of polyunsaturated fatty acids in cell membranes that generates free radicals, which can alter membrane permeability, fluidity, and integrity. Although the generation of free radicals is a continuous physiological process, excessive production can lead to oxidative lesions^39^.

Regarding protein oxidation, the method used to quantify carbonyl groups in *A. aegypti* larvae indicates a direct reaction of the proteins with ROS^40^; when the proteins go through the oxidative process, derivatives or fragments of peptides containing carbonyl groups are formed^40^. Thus, the presence of these peptides indicates cellular damage^41^. 

These results are consistent with those reported by Ahmed^18^ and Zhang et al.^42^. Ahmed focused on lipid peroxidation and protein oxidation as important biomarkers of cell collapse after infection by microorganisms in larvae and adult mosquitoes of *A. caspius* (Culicidae) and found that oxidative stress and cytotoxicity led to lipid membrane damage, mitochondrial dysfunction, and cell death^18^. Zhang et al., however, found that α-terthienyl showed larvicidal activity at a concentration of 0.27 mg.L^-1^ and increased mitochondrial ROS levels in *A. aegypti* larvae. This increase in ROS levels led to mitochondrial dysfunction, organelle damage, and accelerated cell death in larvae treated with α-terthienyl.

Taken together, the results of the present and previous studies suggest that the death of larvae treated with *Xylaria* sp. extract is related to increased oxidative stress, as evidenced by the production of peroxides, MDA, and carbonylated proteins. It is noteworthy that oxidative damage caused by endophytic fungi in *A. aegypti* larvae has not been reported, as previous reports only contain data on plants, algae, and bacteria. However, further studies on the death mechanisms of larvae/insects exposed to fungal extracts may provide valuable insights into the safe control of these vectors.

## References

[1] Brasil M da S (2017). Boletim Epidemiológico.

[2] Costa IMP, Calado DC (2016). Incidência dos casos de dengue (2007-2013) e distribuição sazonal de culicídeos (2012-2013) em Barreiras, Bahia. Epidemiol e Serv Saude.

[3] BRASIL M da S (2019). Biblioteca Virtual em Saúde do Ministério da Saúde Vigilância em Saúde no Brasil 2003|2019 Boletim Epidemiológico.

[4] Rose RI (2001). Pesticides and public health: Integrated methods of mosquito management. Emerging Infectious Diseases. Centers for Disease Control and Prevention.

[5] Para E, Braga IA, Valle D (2007). Aedes Aegypti: Insecticides, Mechanisms of Action and Resistance Artigo de revisão Aedes aegypti: inseticidas, mecanismos de ação e resistência. Epidemiol Serv Saúde.

[6] Zara ALS, Santos SM, Fernandes-Oliveira ES, Carvalho RG, Coelho GE (2016). Estratégias de controle do Aedes aegypti: uma revisão. Epidemiol Serv Saúde.

[7] Cárdenas A, Orozco PJ, Rodríguez CE, Moneriz PC, Díaz CF, Mendez CDM (2013). Aproximación al estudio del daño oxidativo causado por larvicidas naturales y temefos sobre proteomas de larvas del mosquito Aedes aegypti. Cienc y Salud Virtual.

[8] Tauil PL (2002). Critical aspects of dengue control in Brazil. Cad Saúde Pública.

[9] Penna MLF (2003). A challenge for the public health system in Brazil: dengue control. Cad Saúde Pública.

[10] Barreto ML, Teixeira MG (2008). Dengue no Brasil: Situação epidemiológica e contribuições para uma agenda de pesquisa. Estud Avancados.

[11] Pinheiro JB, Polonio JC, Orlandelli RC, Pamphile JA, Golias HC (2020). Atividade larvicida de fungos endofíticos: uma revisão. Brazilian J Dev.

[12] Costa MBS, de Oliveira CM (2020). Endophytic Fungi In The Fight Against Neglected Tropical Diseases. Mini-Reviews Med Chem.

[13] Ayumi TB, Gabriela BST, Daiane OM, Gustavo BL, Yasuo OJ, Rafaela SRMA (2020). Larvicidal and ovocidal effects of Crotalaria pallida extracts on the vector Aedes aegypti. Brazilian J Dev.

[14] Abutaha N, Mashaly AMA, Al-Mekhlafi FA, Farooq M, Al-shami M, Wadaan MA (2015). Larvicidal activity of endophytic fungal extract of Cochliobolus spicifer (Pleosporales: Pleosporaceae) on Aedes caspius and Culex pipiens (Diptera: Culicidae). Appl Entomol Zool.

[15] Tian J, Liu XC, Liu ZL, Lai D, Zhou L (2016). Larvicidal spirobisnaphthalenes from the endophytic fungus Berkleasmium sp. against Aedes albopictus. Pest Manag Sci.

[16] Marques AM, Kaplan MAC (2015). Active metabolites of the genus Piper against Aedes aegypti : natural alternative sources for dengue vector control. Univ Sci.

[17] Ray S, Singh V, Singh S, Sarma BK, Singh HB (2016). Biochemical and histochemical analyses revealing endophytic Alcaligenes faecalis mediated suppression of oxidative stress in Abelmoschus esculentus challenged with Sclerotium rolfsii. Plant Physiol Biochem.

[18] Ahmed AM (2012). Lipid Peroxidation and Oxidative Protein Products as Biomarkers of Oxidative Stress in the Autogenous Mosquito, Aedes caspius, Upon Infection with the Mosquitocidal Bacterium, Bacillus thuringiensis kurstaki. undefined. Pak J Zool.

[19] Thongwat D, Pimolsri U, Somboon P (2015). Screening for mosquito larvicidal activity of thai mushroom extracts with special reference to steccherinum sp against Aedes aegypti (L.) (Diptera: Culicidae). Southeast Asian J Trop Med Public Health.

[20] Lee SR, Kreuzenbeck NB, Jang M, Oh T, Ko S, Ahn JS (2020). Xyloneside A: A New Glycosylated Incisterol Derivative from Xylaria sp. FB. ChemBioChem.

[21] Cañón ERP, Albuquerque MP, Alves RP, Pereira AB, Victoria FDC (2019). Morphological and molecular characterization of three endolichenic isolates of Xylaria (Xylariaceae), from cladonia curta ahti & marcelli (cladoniaceae). Plants.

[22] Ibrahim A, Tanney JB, Fei F, Seifert KA, Cutler GC, Capretta A (2020). Metabolomic-guided discovery of cyclic nonribosomal peptides from Xylaria ellisii sp. nov., a leaf and stem endophyte of Vaccinium angustifolium. Sci Rep.

[23] Maier W, Hammer K, Dammann U, Schulz B, Strack D (1997). Accumulation of sesquiterpenoid cyclohexenone derivatives induced by an arbuscular mycorrhizal fungus in members of the Poaceae. Planta.

[24] Queiroz FMM, Oliveira KW, Cunha MMF, Schwarz A (2013). Evaluation of (anti)genotoxic activities of Phyllanthus niruri L. in rat bone marrow using the micronucleus test. Braz J Pharm Sci.

[25] Simões SS, Costa MBS, Souza AQL, Sousa WC, Oliveira CM (2020). Brazilian Applied Science Review. Braz Ap Sci Rev.

[26] Medeiros E, Rodrigues IB, Litaiff-Abreu E, Da AC, Pinto S, Tadei WP (2013). Larvicidal activity of clove (Eugenia caryophyllata) extracts and eugenol against Aedes aegypti and Anopheles darlingi. African J Biotechnol.

[27] Robertson JL, Preisler HK, Russell RM (2003). Polo Plus Probit and Logit Analysis, User’s Guide.

[28] Ansar Ahmed S, Gogal RM, Walsh JE (1994). A new rapid and simple non-radioactive assay to monitor and determine the proliferation of lymphocytes: an alternative to [3H]thymidine incorporation assay. J Immunol Methods.

[29] Eruslanov E, Kusmartsev S (2010). Identification of ROS using oxidized DCFDA and flow-cytometry. Methods Mol Biol.

[30] Sofic E, Sapcanin A, Tahirovic I, Gavrankapetanovic I, Jellinger K, Reynolds GP (2006). Antioxidant capacity in postmortem brain tissues of Parkinson’s and Alzheimer’s diseases. J Neural Transm Suppl.

[31] Ohkawa H, Ohishi N, Yagi K (1979). Assay for lipid peroxides in animal tissues by thiobarbituric acid reaction. Anal Biochem.

[32] Levine RL, Garland D, Oliver CN, Amici A, Climent I, Lenz AG (1990). Determination of Carbonyl Content in Oxidatively Modified Proteins. Methods Enzymol.

[33] Lowry OH, Rosebrough NJ, Farr AL, Randall RJ (1951). Protein measurement with the Folin phenol reagent. J Biol Chem.

[34] Organização Mundial da Saúde - OMS (2013). Orientações técnica para utilização do larvicida pyriproxyfen (0,5 G) no controle de *Aedes aegypti*.

[35] Zhao J, Shan T, Mou Y, Zhou L (2011). Plant-Derived Bioactive Compounds Produced by Endophytic Fungi. Mini-Reviews Med Chem.

[36] Sánchez-Ortiz BL, Sánchez-Fernández RE, Duarte G, Lappe-Oliveras P, Macías-Rubalcava ML (2016). Antifungal, anti-oomycete and phytotoxic effects of volatile organic compounds from the endophytic fungus Xylaria sp. strain PB3f3 isolated from Haematoxylon brasiletto. J Appl Microbiol.

[37] Bücker A, Bücker NCF, Souza AQL, Gama AM, Rodrigues-Filho E, Costa FM (2013). Larvicidal effects of endophytic and basidiomycete fungus extracts on Aedes and Anopheles larvae (Diptera, Culicidae). Rev Soc Bras Med Trop.

[38] Moro AM, Brucker N, Charão M, Bulcão R, Freitas F, Baierle M (2012). Evaluation of genotoxicity and oxidative damage in painters exposed to low levels of toluene. Mutat Res - Genet Toxicol Environ Mutagen.

[39] Barbosa KBF, Costa NMB, Alfenas CGR, Paula SO, Minim VPR, Bressan J (2010). Estresse oxidativo: Conceito, implicações e fatores modulatórios. Revista de Nutrição.

[40] Stadtman ER, Levine RL (2003). Free radical-mediated oxidation of free amino acids and amino acid residues in proteins. Amino Acids. Amino Acids.

[41] Cong S, Dong W, Zhao J, Hu R, Long Y, Chi X (2020). Characterization of the Lipid Oxidation Process of Robusta Green Coffee Beans and Shelf Life Prediction during Accelerated Storage. Molecules.

[42] Zhang J, Ahmad S, Wang LY, Han Q, Zhang JC, Luo YP (2019). Cell death induced by α-terthienyl via reactive oxygen species-mediated mitochondrial dysfunction and oxidative stress in the midgut of Aedes aegypti larvae. Free Radic Biol Med.

